# Preoperative pelvic MRI and 2-[^18^F]FDG PET/CT for lymph node staging and prognostication in endometrial cancer—time to revisit current imaging guidelines?

**DOI:** 10.1007/s00330-022-08949-3

**Published:** 2022-06-28

**Authors:** Kristine E. Fasmer, Ankush Gulati, Julie A. Dybvik, Kari S. Wagner-Larsen, Njål Lura, Øyvind Salvesen, David Forsse, Jone Trovik, Johanna M. A. Pijnenborg, Camilla Krakstad, Ingfrid S. Haldorsen

**Affiliations:** 1 Mohn Medical Imaging and Visualization Centre (MMIV), Department of Radiology, Haukeland University Hospital, Bergen, Norway; 2 Section for Radiology, Department of Clinical Medicine, University of Bergen, Bergen, Norway; 3 Unit for applied Clinical Research, Department of Public Health and Nursing, Norwegian University of Science and Technology, Trondheim, Norway; 4 Department of Obstetrics and Gynaecology, Haukeland University Hospital, Bergen, Norway; 5 Centre for Cancer Biomarkers, Department of Clinical Science, University of Bergen, Bergen, Norway; 6 Department of Obstetrics and Gynaecology, Radboud University Medical Center, Nijmegen, The Netherlands

**Keywords:** Endometrial neoplasms, Magnetic resonance imaging, Fluorodeoxyglucose F18, Positron emission tomography computed tomography, Neoplasm staging

## Abstract

**Objective:**

This study presents the diagnostic performance of four different preoperative imaging workups (IWs) for prediction of lymph node metastases (LNMs) in endometrial cancer (EC): pelvic MRI alone (IW1), MRI and [^18^F]FDG-PET/CT in all patients (IW2), MRI with selective [^18^F]FDG-PET/CT if high-risk preoperative histology (IW3), and MRI with selective [^18^F]FDG-PET/CT if MRI indicates FIGO stage ≥ 1B (IW4).

**Methods:**

In 361 EC patients, preoperative staging parameters from both pelvic MRI and [^18^F]FDG-PET/CT were recorded. Area under receiver operating characteristic curves (ROC AUC) compared the diagnostic performance for the different imaging parameters and workups for predicting surgicopathological FIGO stage. Survival data were assessed using Kaplan-Meier estimator with log-rank test.

**Results:**

MRI and [^18^F]FDG-PET/CT staging parameters yielded similar AUCs for predicting corresponding FIGO staging parameters in low-risk versus high-risk histology groups (*p* ≥ 0.16). The sensitivities, specificities, and AUCs for LNM prediction were as follows: IW1—33% [9/27], 95% [185/193], and 0.64; IW2—56% [15/27], 90% [174/193], and 0.73 (*p* = 0.04 vs. IW1); IW3—44% [12/27], 94% [181/193], and 0.69 (*p* = 0.13 vs. IW1); and IW4—52% [14/27], 91% [176/193], and 0.72 (*p* = 0.06 vs. IW1). IW3 and IW4 selected 34% [121/361] and 54% [194/361] to [^18^F]FDG-PET/CT, respectively. Employing IW4 identified three distinct patient risk groups that exhibited increasing FIGO stage (*p *< 0.001) and stepwise reductions in survival (*p* ≤ 0.002).

**Conclusion:**

Selective [^18^F]FDG-PET/CT in patients with high-risk MRI findings yields better detection of LNM than MRI alone, and similar diagnostic performance to that of MRI and [^18^F]FDG-PET/CT in all.

**Key Points:**

• *Imaging by MRI and [*^*18*^*F]FDG PET/CT yields similar diagnostic performance in low- and high-risk histology groups for predicting central FIGO staging parameters.*

• *Utilizing a stepwise imaging workup with MRI in all patients and [*^*18*^*F]FDG-PET/CT in selected patients based on MRI findings identifies preoperative risk groups exhibiting significantly different survival.*

• *The proposed imaging workup selecting ~54% of the patients to [*^*18*^*F]FDG-PET/CT yield better detection of LNMs than MRI alone, and similar LNM detection to that of MRI and [*^*18*^*F]FDG-PET/CT in all.*

**Supplementary Information:**

The online version contains supplementary material available at 10.1007/s00330-022-08949-3.

## Introduction

Endometrial cancer (EC) is one of the most common gynecologic malignancy in high-income countries and the incidence has been rising during the last decades [1, 2]. Most women are diagnosed with localized disease, typically indicating favorable prognosis (5-year survival rates > 95%). However, the high number of new cases (estimated 411,367 annually, worldwide) and the fact that women with advanced disease face less favorable prognosis (5-year survival rates down to 20%) [1, 3, 4] motivate the development of safe and effective diagnostic workups for EC patients.

Standard primary treatment of EC is hysterectomy with bilateral salpingo-oophorectomy allowing surgicopathological staging according to the FIGO system [5]. Depending on presumed risk of advanced stage, recommended surgical lymph node (LN) staging strategy ranges from no LN sampling to sentinel LN mapping (SLN), or lymphadenectomy with resection of pelvic and/or para-aortic LNs [6–8]. Prior to surgery, an endometrial biopsy is typically evaluated and classified into low risk (endometrioid grades 1–2), or high risk (endometrioid grade 3 and non-endometrioid) [6, 7]. However, there are limitations in accuracies for histopathologic evaluation based on biopsies [9, 10]. Furthermore, although preoperative high-risk histology is associated with tumor extension and LN metastases (LNM), 8–10% of low-risk EC still presents with LNM [11].

Pelvic imaging by MRI has over the past decades become widely used for non-invasive preoperative local staging of EC [12–14]. Whole-body 2-[^18^F]fluoro-2-deoxy-d-glucose (FDG)-PET/CT has also proven to be useful, especially for detecting LNM and distant spread [15–17]. Although some EC guidelines state that preoperative FDG-PET/CT may be appropriate in patients with high-risk clinical and histologic features [6, 7, 18], FDG-PET/CT is costly and not routinely performed at most centers. However, no studies have systematically investigated the diagnostic value of MRI and FDG-PET/CT in patients with preoperative low-risk versus high-risk histology, nor aimed to define an optimal preoperative imaging workup using MRI combined with selective FDG-PET/CT in EC patients.

In the present study, we hence aim to evaluate the diagnostic performance of MRI and FDG-PET/CT for local staging in EC patients with preoperative low- vs. high-risk histology. In addition, we aim to compare the diagnostic performance for predicting LNM, using four different imaging workups (IWs): MRI in all (IW1), both MRI and FDG-PET/CT in all (IW2), MRI with selective FDG-PET/CT in cases with preoperative high-risk histology (IW3) or MRI with selective FDG-PET/CT in cases with high-risk MRI findings (IW4).

## Materials and methods

### Patient cohort

This retrospective study included 361 consecutive EC patients diagnosed and treated from October 2011 to July 2019 at the same university hospital (serving a population of ~1 million inhabitants). The diagnosis was established through preoperative biopsy, and histologically verified in hysterectomy specimen. All included patients underwent both pelvic MRI and whole-body FDG-PET/CT with standardized protocols (Table 1) prior to treatment. The images were retrospectively evaluated by radiologists/nuclear medicine physicians who were blinded to clinical information and the original imaging reports. Reference standard was surgicopathological FIGO 2009 stage [5]. Patients diagnosed at our institution during October 2011 to July 2019 that did not have preoperative standardized MRI and/or FDG-PET/CT (*n* = 253), or with missing preoperative histology status (*n* = 11), were not included in the study (supplementary Table S1). This study was conducted under Institutional Review Board (IRB)-approved protocols (IRB approvals 2015/2333; 2015/548; biobank approval 2014/1907) with written informed consent from all patients at primary diagnosis. The consent included approval of prospective collection of clinical patient data, tissue samples, and imaging data (e.g., CT, MRI, PET/CT) from primary diagnostic workup and during follow-ups.

**Table 1:** Pelvic MRI and whole-body [^18^F]FDG-PET/CT acquisition and reconstruction parameters. All scanners are from Siemens

Pelvic MRI:						
MRI	Sequence	Plane	TR/TE_1_/TE_2_ (ms)	FA (Deg)	FOV (mm)	Voxel size (mm)
1.5T Avanto	T2 TSE	AO	6310/95	150	180 × 180	0.9 × 0.7 × 3.0
	T2 TSE	SAG	4920/95	150	180 × 180	0.9 × 0.7 × 3.0
	DWI	AO	3100/79	90	300 × 300	2.3 × 2.3 × 5.0
	T1 VIBE	AO	7.2/2.6	20	250 × 250	1.6 × 1.3 × 2.0
3T Skyra	T2 TSE	AO	4330/94	150	200 × 200	0.5 × 0.5 × 3.0
	T2 TSE	SAG	7360/101	160	200 × 200	0.6 × 0.6﻿ × 3.0
	DWI RESOLVE	AO	6010/74/126	180	200 × 200	1.4﻿ × 1.4 × 3.0
	T1 DIXON	AO	5.9/2.5/3.7	9	250 × 250	1.0 × 1.0 × 1.2
Whole-body [^18^F]FDG-PET/CT:						
PET	Scan time/speed	Corrections	Iteration/subset	Filter	FOV (mm)	Voxel size (mm)
Truepoint	3 min/bed	ATTN/SCAT	4/8	5 mm gauss	700	4.1 × 4.1 × 5.0
Vision	1.1 mm/s	ATTN/SCAT/PSF/TOF	4/5	All pass	700	1.7 × 1.7 × 3.0
CT	CareDose (ref mAs)	CarekV (kV)	Recon	Filter/IR strength	FOV scan/recon (mm)	Voxel size (mm)
Truepoint	Yes (50/240)^a^	No (120)	FB	B19f	500/700	1.4 × 1.4 × 5.0
Vision	Yes (25/210)^a^	Yes (120)	IR	I30f/5	500/780	1.5 × 1.5 × 3.0

*AO* axial oblique slice orientation, *ATTN* attenuation correction, Deg degrees, *DWI* diffusion weighted imaging, *FA* flip angle, *FB* filtered back projection reconstruction, *FOV* field of view, *IR* iterative reconstruction, *PSF* point spread function correction, *RESOLVE* readout segmentation of long variable echo trains, *SCAT* scatter correction, *TE* time echo, *TOF* time of flight correction, *TR* repetition time, *TSE* turbo spin echo, *VIBE* volumetric interpolated breath-hold examination

^a^Reference mAs for low-dose/diagnostic CT protocols

Preoperative endometrial biopsies by pipelle or curettage were histologically classified as endometrioid grades 1–3 (EEC G1–G3), or non-endometrioid endometrial carcinoma (NEEC). Pelvic lymphadenectomy with/without para-aortic lymphadenectomy was routinely performed in all patients with preoperatively defined high-risk disease (NEEC or EEC G3 histology with deep (≥ 50%) myometrial invasion assessed by MRI (MI_MRI_)) according to the European Society of Medical Oncology (ESMO) guidelines [8]. For patients with preoperative low-risk_ESMO_ (EEC G1–G2 with MI_MRI_ < 50%) and intermediate-risk_ESMO_ (EEC G1–G2 with MI_MRI_ ≥ 50%, or EEC G3 with MI_MRI_ < 50%), pelvic lymphadenectomy was selectively omitted if negative imaging findings for LNM and if preserved tumor hormone receptor status (see [19] for further details). Surgical specimens were assessed by pathologists, reporting presence of deep myometrial invasion (MI ≥ 50%), cervical stroma invasion (CI), LNM, and tumor histologic subtype and grade (EEC G1–G3 or NEEC) [20]. Patients were surgically staged according to the FIGO 2009 criteria [5].

Clinical and patient follow-up data were collected from medical records (last accessed 14 September 2021). Progression was defined as local recurrence or progression in the pelvis or new metastases in the abdomen or at distant locations.

### MRI scanning

**Fig. 1:**
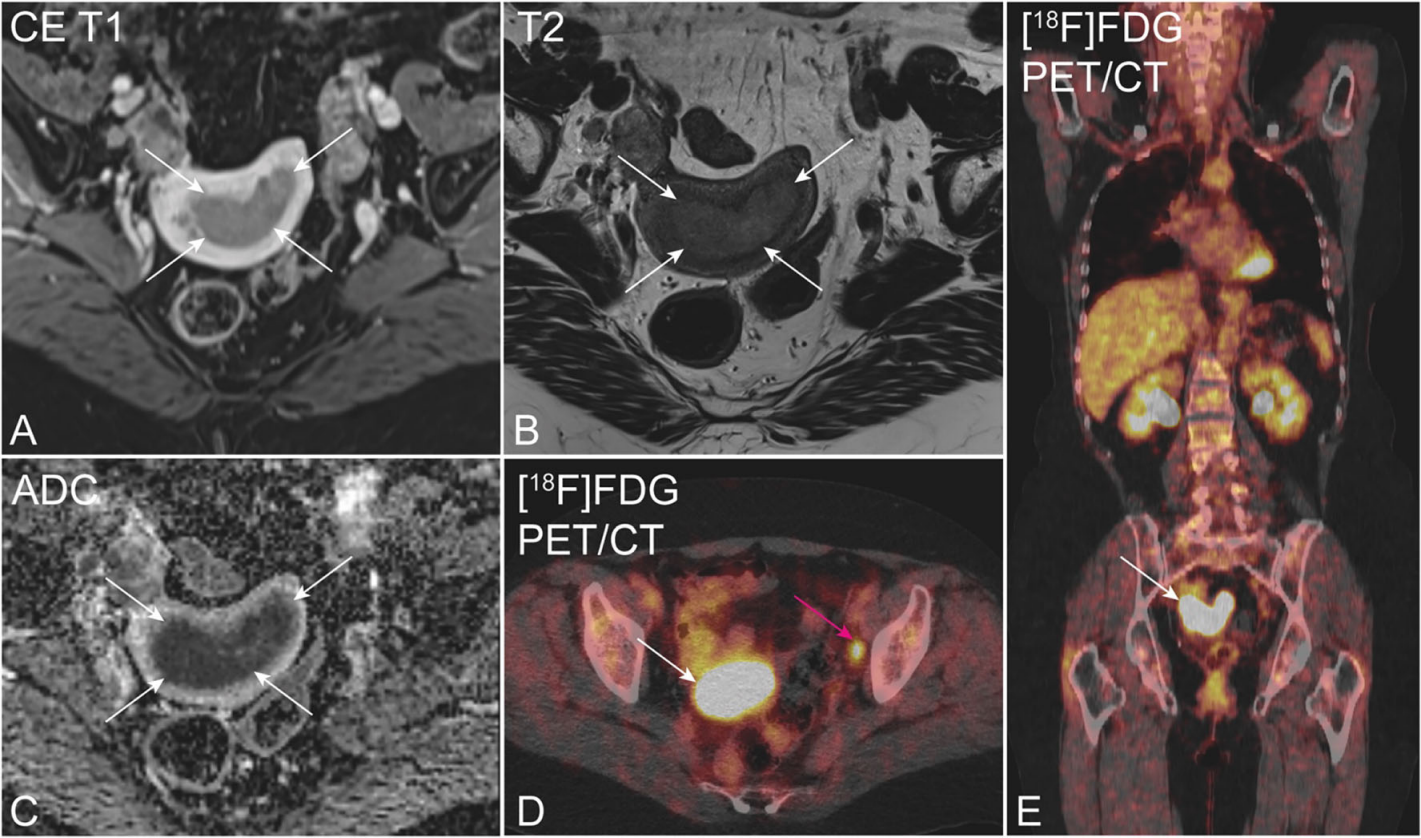
Preoperative pelvic MRI (**A**–**C**) and [^18^F]FDG PET/CT (**D**, **E**) in a 72-year-old patient with endometrial carcinoma, FIGO stage IIIC (endometrioid, grade G1). Axial oblique contrast-enhanced T1-weighted (**A**) and T2-weighted MRI (**B**) depicts an arcuately shaped uterine tumor (white arrows), exhibiting restricted diffusion at apparent diffusion coefficient map (**C**). Axial (**D**) and coronal (**E**) [^18^F]FDG PET/CT shows elevated [^18^F]FDG uptake both in the primary tumor (white arrow) and in a left iliac lymph node (cerise arrow)

Preoperative pelvic MRI was acquired on a 1.5-T Avanto scanner for 157/361 patients, and on a 3-T Skyra scanner for the remaining 204/361 patients (both Siemens). Prior to imaging, patients were given butylscopolamine bromide intramuscularly/intravenously (20 mg Buscopan, Boehringer Ingelheim), in order to reduce bowel peristalsis. The MRI protocols consisted of contrast-enhanced (CE) axial oblique (perpendicular to the long axis of the uterine body) T1-weighted gradient echo volumetric interpolated breath-hold (VIBE) (1.5T), and VIBE DIXON (3T) images, acquired before and 2 min after administration of contrast agent (0.1 mmol gadolinium/kg body weight) (Fig. 1). Also included in the protocol were sagittal and axial oblique T2-weighted turbo spin echo images, and axial oblique diffusion-weighted images (DWI) with *b*-values of 0 and 1000 s/mm^2^ (1.5T) or 0, 500, and 1000 s/mm^2^ (3T). Protocols at both field strengths (Table 1) were in line with guidelines from the European Society of Urogenital Imaging [12, 13].

### MRI reading

All MRI images were de-identified and read independently in PACS by three different readers—in total, eight radiologists with 2–10 years of experience with pelvic MRI. The radiologists were blinded to clinical, pathological, and patient outcome data as well as the MRI report and FDG-PET/CT findings. Myometrial invasion (MI_MRI_: </≥ 50%), cervical stroma invasion (CI_MRI_: yes/no), and enlarged para-aortic or para-iliac LNs (largest LN short-axis diameter LN_MRI_: </≥ 10 mm, irrespective of plane) were recorded separately by all three readers on standardized MRI registration forms. Consensus values were established using the category recorded by the majority of the three readers. Although not reported in the present study, DWI and apparent diffusion coefficient maps were available for the radiologists for visual inspection of (restricted) diffusion in the primary tumor and metastases.

### FDG-PET/CT scanning

Preoperative FDG-PET/CT was acquired on a Biograph TruePoint for 325/361 patients and on a Biograph Vision scanner for the remaining 36/361 patients (both Siemens). All patients were instructed to fast for 6 h prior to scanning and FDG was given intravenously approximately 60 min prior to scanning (TruePoint: 370 MBq or 4.6 MBq/kg, Vision: 3 MBq/kg). PET/CT images were acquired from skull base to mid-thigh and PET images were corrected for attenuation and scatter using the CT scan. Further details on PET/CT acquisition and reconstruction are given in Table 1.

### FDG-PET/CT reading

All PET images were reviewed on an Oasis workstation (Segami Corporation) by one reader—in total, two nuclear medicine physicians, both with > 4 years of FDG-PET/CT experience. The reader was blinded to clinical, pathological, and patient outcome data as well as the FDG-PET/CT report and MRI findings. Increased FDG uptake (standardized uptake value (SUV) > 2.5) in primary tumor, lymph nodes (LN_PET_: yes/no), and suspected distant metastases (Fig. 1) were recorded in standardized PET registration forms. Finally, PET and the consensus MRI reading were merged with clinical, surgicopathological, and outcome data for further analyses.

### Preoperative EC imaging workups (IW1-4) and preoperative risk classification (low, intermediate, and high risk) based on IW4

In order to compare the diagnostic performance metrics for prediction of LNM in EC, four different imaging workups were constructed: IW1, MRI in all patients; IW2, both MRI and FDG-PET/CT in all patients; IW3, MRI with selective FDG-PET/CT in patients with preoperative EEC G3 or NEEC histology; and IW4, MRI with selective FDG-PET/CT in patients with either MI_MRI_ ≥ 50%, CI_MRI_, or enlarged LN_MRI_. Detection of LNM were based on either LN_MRI_ alone (IW1) or LN_MRI_ combined with LN_PET_ (IW2-4). Only patients with surgical LN staging (*n* = 220) were included in these analyses (Table 2, supplementary Table S2).

**Table 2:** Clinical and surgicopathological characteristics in 361 EC patients who underwent preoperative pelvic MRI and [^18^F]FDG PET/CT and had preoperative histology from biopsy/curettage

Age (median, range)	68 (30, 90)
Preoperative histology^a^, *n* (%)	
EEC G1–G2 (low-risk)	240 (66)
EEC G3+NEEC (high-risk)	121 (34)
MI^b^, *n* (%)	
< 50%	212 (60)
≥ 50%	144 (40)
CI^b^, *n* (%)	
No	308 (86)
Yes	51 (14)
Lymph node surgery, *n* (%)	
No	141 (39)
Yes^c^	220 (61)
LNM^b^, *n* (%)	
No	193 (88)
Yes	27 (12)
Histologic subtype^b^, *n* (%)	
EEC (G1–G3)	285 (79)
NEEC	76 (21)
Histologic grade (EEC only)^b^, *n* (%)	
G1–G2	240 (85)
G3	43 (15)
FIGO stage^b^, *n* (%)	
I	283 (78)
II	33 (9)
III	34 (9)
IV	11 (3)
Adjuvant therapy, *n* (%)	
No	230 (64)
Yes^d^	131 (36)

*CI* cervical stroma invasion, *MI* myometrial invasion, *EEC* endometrioid endometrial carcinoma, *G* grade, *NEEC* non-endometrioid endometrial carcinoma, *FIGO* The International Federation of Gynecology and Obstetrics System, *LNM* lymph node metastases

^a^Based on preoperative biopsy from curettage/pipelle

^b^Based on final histopathology after surgical staging. Missing data (numbers): MI (5), CI (2), histologic grade (2)

^c^Pelvic lymph node sampling in 220/361 patients with accompanying paraaortic lymph node sampling in 79/361 patients

^d^Chemotherapy (*n* = 123), external radiation therapy (*n* = 3), hormonal treatment (*n* = 3), and brachytherapy (*n* = 2).

**Fig. 2:**
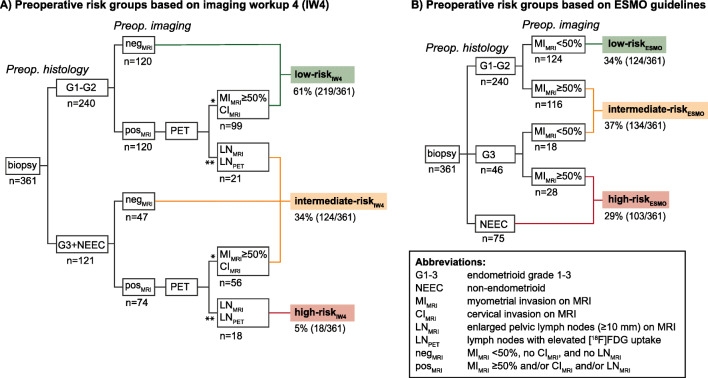
Preoperative risk groups based on (**A**) imaging workup 4 (IW4), combining preoperative histology and imaging results from MRI and from selective [^18^F]FDG PET/CT based on MRI findings, and (**B**) the ESMO 2013 clinical practice guidelines for endometrial cancer (EC) in the same EC cohort (*n* = 361). Applying IW4 stratifies 61% of the patients as low-risk, 34% as intermediate-risk, and 5% as high-risk (**A**), whereas the ESMO guidelines stratifies 34% of the patients as low-risk, 37% as intermediate-risk, and 29% as high-risk (**B**). Preoperative histology from curettage/pipelle are classified as endometrioid grades 1–2 (G1–G2), endometrioid grade 3 (G3) or non-endometrioid (NEEC). *This subgroup includes patients with either MI_MRI_ ≥ 50% or CI_MRI_, but negative lymph node findings on both MRI and [^18^F]FDG PET/CT. **This subgroup includes patients with positive lymph node findings on MRI and/or [^18^F]FDG PET/CT

Applying imaging findings based on IW4, we identified patient groups (Fig. 2A) exhibiting different LNM prevalence (supplementary Figure S1) and survival (supplementary Figure S2), and compared the patient risk groups from IW4 with that based in the ESMO risk classification [8] (Fig. 2B). Using IW4, the low-risk_IW4_ group was defined as preoperative EEC G1–G2 histology with either negative MRI findings (for patients with MRI only), or negative LN findings on both MRI and FDG-PET/CT (for patients with both modalities). The intermediate-risk_IW4_ group was defined as either preoperative EEC G1–G2 histology with LN_MRI_ and/or LN_PET_, or preoperative EEC G3+NEEC histology with either negative MRI findings (for patients with MRI only), or negative LN findings on both MRI and FDG-PET/CT. The high-risk_IW4_ group was defined as preoperative EEC G3+NEEC histology with LN_MRI_ and/or LN_PET_ (Fig. 2A).

### Statistical analyses

Receiver operating characteristic (ROC) analyses were employed to compare the diagnostic performance of the imaging parameters MI_MRI_, CI_MRI_, LN_MRI_, and LN_PET_ for prediction of surgically assessed MI, CI, and LNM. ROC analyses were also used to assess the different imaging workups’ (IW1–4) performance for predicting histologically confirmed LNM. Area under ROC curves (AUCs) were compared using DeLong’s test of equality. The Kruskal-Wallis test (with ties) was used to analyze the proposed preoperative risk groups based on IW4 in relation to surgicopathological staging results. The prognostic value of preoperative risk groups based on IW4 and ESMO guidelines was explored using Kaplan-Meier estimator with log-rank test. All statistical analyses were performed with Stata 17.0 (StataCorp).

## Results

### Patients and treatment

During October 2011 to July 2019, a population-based endometrial cancer cohort of 625 consenting patients was prospectively collected at our institution. Of these, 361 patients with preoperative standardized MRI and/or FDG-PET/CT were included in this study, while 264 patients lacking standardized MRI and/or FDG-PET/CT (*n* = 253), or without preoperative histology status (*n* = 11), were excluded. Clinical and surgicopathological characteristics for all included patients (*n* = 361) are given in Table 2. Quite similar surgicopathological patient characteristics were observed in the study cohort (*n *= 361) and the entire patient cohort (*n* = 625, supplementary Table S1).

Primary treatment consisted of hysterectomy with bilateral salpingo-oophorectomy in 98% (355/361) of the patients. These patients were all surgically staged according to FIGO 2009 staging system (reference standard) [5]. The remaining patients either underwent fertility-sparing treatment (*n* = 1, presumed FIGO I) or tumor debulking surgery (*n* = 1, presumed FIGO IV) or were assessed ineligible for surgery (*n* = 4, presumed FIGO IV). The presumed FIGO stage was based on results from preoperative/operative specimen and diagnostic workup. Pelvic LN surgery was performed in 61% [220/361] of the patients, and 22% [79/361] had accompanying para-aortic lymphadenectomy. Lymphadenectomy was, as expected, more often performed in patients with high-risk preoperative histology (87% [105/121]) compared with patients with low-risk preoperative histology (48% [115/240], supplementary Table S3). Median (range) time span between preoperative imaging and surgical staging was 16 [0–98] days for MRI and 16 [0–89] days for FDG-PET/CT. Median (range) follow-up time was 53 [3–106] months.

### MRI and FDG-PET/CT for prediction of surgicopathological MI, CI, and LNM in patients with low-risk vs. high-risk preoperative histology

Diagnostic performance metrics of MI_MRI_, CI_MRI_, LN_MRI_, and LN_PET_ for predicting histopathological MI, CI, and LNM after surgical staging are given in Table 3 for patients with low-risk (EEC G1–G2) and high-risk (EEC G3+NEEC) preoperative histology, respectively. As shown by the similarity in ROC AUC, there were no overall differences in diagnostic performance metrics based on positive MRI and PET findings in patients with low- vs high-risk preoperative histology (*p* ≥ 0.16 for all).

**Table 3:** Performance of preoperative MRI and [^18^F]FDG PET/CT findings for diagnosing histopathological deep myometrial invasion (MI ≥ 50%), cervical stroma invasion (CI) and lymph node metastases (LNM) for patients preoperatively assessed with low-risk (endometrioid G1–G2) (*n* = 240) versus high-risk (endometrioid G3 or non-endometrioid (G3+NEEC)) (*n* = 121) histology (based on biopsy from curettage/pipelle)

Imaging findings^a^	Histopath. findings^b^	Preoperative histology (*n*)	Sensitivity % (*n*)	Specificity % (*n*)	Accuracy % (*n*)	PPV % (*n*)	NPV % (*n*)	ROC AUC (95% ci)	*p*^c^
MI_MRI_ ≥ 50%	MI ≥ 50%	G1–G2 (239)	79 (75/95)	72 (104/144)	75 (179/239)	65 (75/115)	84 (104/124)	0.76 (0.70–0.91)	0.62
		G3+NEEC (117)	82 (40/49)	65 (44/68)	72 (84/117)	63 (40/64)	83 (44/53)	0.73 (0.65–0.81)	
CI_MRI_	CI	G1–G2 (239)	28 (7/25)	98 (209/214)	90 (216/239)	58 (7/12)	92 (209/227)	0.63 (0.54–0.72)	0.92
		G3+NEEC (120)	31 (8/26)	94 (88/94)	80 (96/120)	57 (8/14)	83 (88/106)	0.62 (0.53–0.72)	
LN_MRI_	LNM^d^	G1–G2 (115)	40 (4/10)	96 (101/105)	91 (105/115)	50 (4/8)	94 (101/107)	0.68 (0.52–0.84)	0.54
		G3+NEEC (105)	29 (5/17)	94 (83/88)	84 (88/105)	50 (5/10)	87 (83/95)	0.62 (0.50–0.73)	
LN_PET_	LNM^d^	G1–G2 (115)	70 (7/10)	92 (97/105)	90 (104/115)	47 (7/15)	97 (97/100)	0.81 (0.66–0.96)	0.16
		G3+NEEC (105)	41 (7/17)	93 (82/88)	85 (89/105)	54 (7/13)	89 (82/92)	0.67 (0.55–0.80)	

*AUC* area under curve, *ci* confidence interval, *CI* cervical stroma invasion, *MI* myometrial invasion, *FIGO* The International Federation of Gynecology and Obstetrics System, *G* grade, *LNM* lymph node metastases, *LN*_*MRI*_ enlarged (≥ 10 mm) pelvic lymph node(s) on MRI, *LN*_*PET*_ suspicious tracer [^18^F]FDG uptake in lymph node(s), *NEEC* non-endometrioid endometrial carcinoma, *NPV* negative predictive value, *PPV* positive predictive value, *ROC* receiver operating characteristic curve

^a^Imaging findings based on MRI and [^18^F]FDG PET/CT

^b^Histopathological findings based on final histopathologic assessment after surgical staging. Missing data (numbers): MI (5), CI (2)

^c^Equality of areas under the ROC curves (AUCs) are assessed by DeLong test

^d^Pelvic lymph node surgery in 220/361 patients

### Prediction of LNM by the different imaging workups IW1–4

Prediction of LNM by the four different imaging workups IW1–4 is given in Table 4. IW2 and IW4 yielded highest sensitivities (56% and 52%, respectively), while IW1 and IW3 yielded highest specificities for predicting LNM (95% and 94%, respectively). Furthermore, IW2 and IW4 yielded the highest areas under the ROC curves for prediction of LNM (AUC = 0.73 and 0.72, respectively; Table 4), which were significantly higher or tended to be higher than that of IW1 (AUC = 0.64; *p* = 0.04 and 0.06, respectively, Table 4). IW3 yielded an AUC of 0.69, similar to that of IW1 (*p* = 0.13, Table 4). When comparing the workups including FDG-PET/CT in selected or all cases (IW2–4), no statistically significant differences between the AUCs were observed (*p *≥ 0.24).

**Table 4:** Prediction of lymph node metastases (LNM) by different imaging workups utilizing pelvic MRI in all (IW1), MRI and [^18^F]FDG PET/CT in all (IW2), MRI in all and selective [^18^F]FDG PET/CT in patients preoperatively assessed with high-risk histology (endometrioid grade 3 or non-endometrioid based on biopsy from curettage/pipelle) (IW3), and MRI in all and selective [^18^F]FDG PET/CT in patients with high-risk MRI-findings (IW4). Only patients with surgical lymph node staging (*n* = 220) are included in these analyses

Imaging workup	Sensitivity % (*n*)	Specificity % (*n*)	Accuracy % (*n*)	PPV % (*n*)	NPV % (*n*)	ROC AUC (95% ci)	*p*^e^
IW1^a^	33 (9/27)	95 (184/193)	88 (193/220)	50 (9/18)	91 (184/202)	0.64 (0.55–0.74)	
IW2^b^	56 (15/27)	90 (174/193)	86 (189/220)	44 (15/34)	94 (174/186)	0.73 (0.63–0.83)	0.04
IW3^c^	44 (12/27)	94 (181/193)	88 (193/220)	50 (12/24)	92 (181/196)	0.69 (0.59–0.79)	0.13
IW4^d^	52 (14/27)	91 (176/193)	86 (190/220)	45 (14/31)	93 (176/189)	0.72 (0.62–0.81)	0.06

*AUC* area under curve, *ci* confidence interval, *NPV* negative predictive value, *PPV* positive predictive value, *ROC* receiver operating characteristic curve

^a^Prediction of LNM based on enlarged (≥ 10 mm) pelvic lymph node(s) on MRI (LN_MRI_)

^b^Prediction of LNM based on LN_MRI_ and elevated [^18^F]FDG uptake in lymph node(s) (LN_PET_) combined

^c^Prediction of LNM based on LN_MRI_ for patients with low-risk preoperative histology (endometrioid grades 1–2), and LN_MRI_ and LN_PET_ combined for patients with high-risk preoperative histology (endometrioid grade 3 or non-endometrioid)

^d^Prediction of LNM based on either LN_MRI_, or LN_MRI_ and LN_PET_ combined depending on imaging findings on MRI

^e^Equality of AUC between workup 2–4 versus workup 1, assessed by DeLong test

When applying IW4 with selective FDG-PET/CT in patients with high-risk MRI findings, 54% [194/361] would undergo FDG-PET/CT (Fig. 2A), whereas when applying IW3 with selective PET/CT if high-risk preoperative histology, 34% [121/361] of the patients would undergo FDG-PET/CT (Table 2).

### Preoperative EC risk classification based on IW4 compared to ESMO 2013 risk classification

**Fig. 3:**
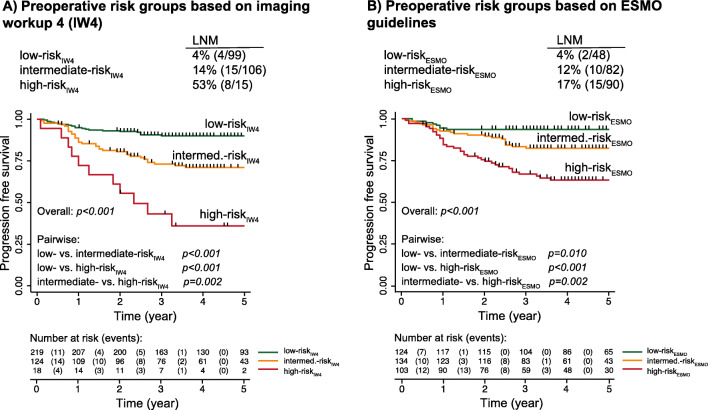
Prevalence of lymph node metastases (LNMs) and Kaplan-Meier curves depicting progression-free survival for the preoperative risk groups based on **A** imaging workup 4 (IW4), combining preoperative histology and imaging results from MRI and from selective [^18^F]FDG PET/CT based on MRI findings, and **B** the ESMO 2013 clinical practice guidelines for endometrial cancer (EC) in the same EC cohort (*n* = 361). *P*-value refers to the Log-Rank test and numbers at risk are given for each time point for the risk groups separately

When using IW4, 61% [219/361] of the patients were classified as low-risk_IW4_, 34% [124/361] as intermediate-risk_IW4_, and 5% [18/361] as high-risk_IW4_ (Fig. 2A). Employing the ESMO risk classification in the same patient cohort yielded a lower proportion of patients in the low-risk_ESMO_ group (34% [124/361], Fig. 2B), while a higher proportion of the patients were classified as intermediate- and high-risk_ESMO_ (37% [134/361] and 29% [103/361], respectively, Fig. 2A). The prevalence of LNM was similar in low-risk_IW4_ vs. low-risk_ESMO_ (4% for both, Fig. 3), and in intermediate-risk_IW4_ vs. intermediate-risk_ESMO_ (14% vs. 12%, Fig. 3), while higher in high-risk_IW4_ compared to high-risk_ESMO_ (53% vs. 17%, Fig. 3).

The low-, intermediate-, and high-risk_IW4_ groups exhibited increasing prevalence of both MI ≥ 50%, CI, LNM, high-grade histology, and advanced FIGO stage (III–IV) (*p *≤ 0.04 for all, Table 5), and a stepwise reduction in progression-free survival (PFS) for the intermediate- and high-risk_IW4_ groups compared with the low-risk_IW4_ group (*p* ≤ 0.002, Fig. 3A). PFS for all of the subgroups within the IW4 categories (Fig. 2A) is presented in supplementary Figure S2.

**Table 5:** Surgicopatological characteristics for the different risk groups defined by the diagnostic workup (IW4) combining preoperative histology and imaging results from MRI and selective [^18^F]FDG PET/CT based on MRI findings in 361 EC patients

Surgicopathological findings^a^ (*n*)	Low-risk_IW4_ % (*n* = 219)	Intermediate-risk_IW4_ % (*n* = 124)	High-risk_IW4_ % (*n* = 18)	*p*^b^
MI (356)				0.04
< 50% (212)	63% (138/218)	56% (69/123)	33% (5/15)	
≥ 50% (144)	37% (80/218)	44% (54/123)	67% (10/15)	
CI (359)				< 0.001
No (308)	92% (200/218)	79% (97/123)	61% (11/18)	
Yes (51)	8% (18/218)	21% (26/123)	39% (7/18)	
LNM^c^ (220)				< 0.001
No (193)	96% (95/99)	86% (91/106)	47% (7/15)	
Yes (27)	4% (4/99)	14% (15/106)	53% (8/15)	
Histologic subtype (361)				< 0.001
EEC (G1–G3) (285)	99% (216/219)	49% (61/124)	44% (8/18)	
NEEC (76)	1% (3/219)	51% (63/124)	56% (10/18)	
Histologic grade (EEC only) (283)				< 0.001
G1–G2 (240)	97% (207/214)	52% (32/61)	12% (1/8)	
G3 (43)	3% (7/214)	48% (29/61)	88% (7/8)	
FIGO (361)				< 0.001
I–II (316)	95% (209/219)	82% (102/124)	28% (5/18)	
III–IV (45)	5% (10/219)	18% (22/124)	72% (13/18)	

*CI* cervical stroma invasion, *MI* myometrial invasion, *EEC* endometrioid endometrial carcinoma, *FIGO* The International Federation of Gynecology and Obstetrics System, *G* grade, *NEEC* non-endometrioid endometrial carcinoma, *LNM* lymph node metastases

^a^Based on final histopathologic assessment at surgical staging

^b^Kruskal-Wallis test with ties

^c^Pelvic lymph node surgery in 220/361 patients

Missing data (numbers): MI (5), CI (2), Grade (2)

## Discussion

This study presents diagnostic performance metrics for central staging parameters by preoperative MRI and FDG-PET/CT in a large EC cohort, showcasing similar diagnostic performance metrics in patients with low-risk versus high-risk preoperative histology. We further demonstrate that a stepwise approach, utilizing preoperative histology results and MRI in all patients, with selective FDG-PET/CT based on MRI findings, allows classification of patients into preoperative risk groups. Importantly, the risk groups exhibit different prevalence of aggressive surgicopathological features (MI, CI, LNM, high grade, and advanced FIGO stage), and significantly different survival. Furthermore, we show that this stepwise approach, selecting ~54% of the patients to FDG-PET/CT, yields better or similar diagnostic performance metrics for diagnosing LNM, compared to diagnostic imaging workups based on MRI alone or on concurrent MRI and FDG-PET/CT in all.

Pelvic MRI has a pivotal role in locoregional staging of EC [6, 7] and multiple studies have shown that CE MRI yields good diagnostic performance, especially for diagnosing MI ≥ 50% [12, 14, 21]. In a meta-analysis by Wu et al [21], CE MRI had a pooled sensitivity for prediction of MI ≥ 50% of 85% (95% CI: 74–93%). Reported interobserver agreement for radiologists reporting MI ≥ 50% is ranging from fair to very good (κ: 0.32–0.84) [22, 23]. In the present study, we found sensitivities for predicting MI ≥ 50% of 79% (low-risk preoperative histology) and 82% (high-risk preoperative histology), both within the range reported by Wu et al. We also showed that the diagnostic performance metrics of MRI for diagnosing MI ≥ 50%, CI, and LNM, were similar in patients with low-risk vs high-risk preoperative histology. Thus, the performance of pelvic MRI seems to be minimally affected by histologic subtype supporting the robustness of MRI for valid preoperative local staging in EC.

The role of FDG-PET/CT in EC imaging is evolving and several studies have shown that FDG-PET/CT compares favorably with conventional MRI for detection of LNM and distant metastases [15, 17, 24, 25]. This is also evident from our study, where MRI only yielded a sensitivity of 33% and an AUC of 0.64 for predicting LNM, while after including FDG-PET/CT in all, the sensitivity and AUC for diagnosing LNM increased to 56% and 0.73, respectively. However, FDG-PET/CT is a costly examination, and access to clinical PET/CT scanning facilities is in many countries limited. Thus, it is important to identify the EC patients that are most likely to benefit from additional FDG-PET/CT as part of their primary diagnostic workup. Several guidelines and previous papers advocate the usage of FDG-PET/CT in patients with presumed high risk of advanced FIGO stage, since these are expected to have the highest probability of LNM and distant spread [6, 16–18]. Oxymoronically, the present study failed to show that selective FDG-PET/CT in high-risk patients based on preoperative histology improves the detection of LNM compared to MRI alone (AUC = 0.69 for IW3 vs. AUC = 0.64 for IW1; *p* = 0.13). It is further evident that including FDG-PET/CT (in addition to MRI) in all patients yields the highest sensitivity (56%) and AUC (0.73) for predicting LNM. However, using an approach with selective FDG-PET/CT based on MRI findings (IW4; sensitivity = 52%, AUC = 0.72) would substantially reduce healthcare costs by only selecting 54% of the patients for FDG-PET/CT, with no significant reduction in diagnostic accuracy compared with that using MRI and FDG-PET/CT in all (IW2).

LN status is one of the main prognostic factors in EC and guides adjuvant treatment [6, 7, 26]. Routine lymphadenectomy in early-stage EC has not been documented to improve outcome and is associated with major comorbidities including lymphedema, lymphocyst formation, and genitofemoral nerve injury [27, 28]. Over the past years, SLN mapping has been introduced as a less-invasive alternative to standard lymphadenectomy in the surgical management of EC. However, SLN mapping requires high surgical expertise, and in a recent survey among gynecological oncologists in Europe and USA, only 50% reported that they used SLN techniques [29]. SLN sampling is also reported to be very challenging in obese patients [30], and leads to increased surgical procedure time and cost compared with that of surgery without lymph node sampling [31]. Thus, preoperative identification of LNM is still essential for optimizing surgical LN staging in patients with LNM, and for safely avoiding invasive staging procedures in patients who do not have LNM (being particularly favorable for patients with comorbidities). Interestingly, the proposed preoperative risk classification using IW4 stratified a higher proportion of patients to low-risk groups (61%) compared to that using the ESMO 2013 classification (34%), however with similar prevalence of LNM (4%) in the low-risk_IW4_ and low-risk_ESMO_ groups. Furthermore, the high-risk_IW4_ group had worse outcome and a higher prevalence of LNM (53%), compared to the high-risk_ESMO_ group (17%).

Several previous EC studies have aimed at predicting LNM by utilizing preoperative molecular biomarkers [11, 32, 33], and a few have also incorporated preoperative imaging findings [34–38]. Reijnen et al have presented a Bayesian network (ENDORISK) that includes molecular and clinical biomarkers, including lymphadenopathy on imaging, yielding AUCs of 0.82–0.84 for predicting LNM [38]. In a study by Berg et al, tumor size measured by MRI was incorporated in a model together with clinical and tumor protein variables, resulting in an AUC of 0.83 for LNM prediction. In the present study, including only preoperative histology and standard imaging findings, the proposed IW4 resulted in a slightly lower AUC of 0.72 for LNM prediction. Although all of the imaging workups (IW1–4) yielded high specificities (90–95%) and NPVs (91–94%) for LNM detection, their relatively low sensitivities (33–56%) and PPVs (44–50%) show that conventional staging information based on preoperative EC imaging has clear limitations. However, several promising functional and structural imaging markers have been reported in EC that may be combined with conventional imaging markers to improve the staging performance of MRI and FDG-PET/CT [14]. For MRI, tumor markers from diffusion-weighted imaging (DWI) [23, 39], dynamic contrast-enhanced MRI [40], tumor size/volume [15, 41], and MRI radiomics [42, 43] have all been reported to predict surgicopathological stage and outcome in EC. For FDG-PET/CT, large metabolic tumor volume [15, 44] and specific PET radiomics features [45] have also been linked to markers of aggressive disease and poor outcome in EC. How information from histological, molecular, and clinical examinations should be combined with tumor information retrieved from conventional and advanced diagnostic imaging in order to yield even better prediction of LNM and outcome in EC should be further explored and validated in independent patient cohorts in future studies.

### Limitations

Lymphadenectomy is, at most sites, omitted in patients with clinical and histological low risk of LNM in order to limit post-surgical side effects. In the present study, lymphadenectomy was performed in only 48% of patients with low-risk preoperative histology versus 87% of the patients with high-risk preoperative histology. This may potentially have introduced an inherent selection bias in the analyses of diagnostic performance metrics for detecting LNM among patients subjected to lymphadenectomy (*n* = 220). This limitation is, however, hard to avoid, as it would be considered unethical to perform LN sampling routinely in low-risk patients who would be unlikely to benefit from this invasive surgical procedure.

## Conclusion

In this large EC study, we evaluated the diagnostic performance metrics for central staging parameters by preoperative MRI and FDG-PET/CT in subgroups with low-risk versus high-risk preoperative histology—finding similar diagnostic performance in the low- and high-risk histology groups. Furthermore, we propose a stepwise imaging workup incorporating information about preoperative MRI findings that selected ~54% of the patients to FDG-PET/CT. This workup yielded high diagnostic performance for prediction of LNM and identified preoperative EC risk groups exhibiting significantly different risks of advanced disease and poor outcome. The proposed stepwise imaging workup represents a promising approach to ensure preoperative image-guided risk stratification and LNM detection in EC, while avoiding unnecessary use of FDG-PET/CT in patients who are less likely to benefit from a costly FDG-PET/CT examination.

## Supplementary Information

ESM 1 (DOCX 442 kb)

## Abbreviations

CI: Cervical stroma invasion
EC: Endometrial cancer
EEC: Endometrioid endometrial carcinoma
ESMO: European Society for Medical Oncology
FDG: 2-[^18^F]Fluoro-2-deoxy-d-glucose
FIGO: The International Federation of Gynecology and Obstetrics system
LNM: Lymph node metastasis
MI: Myometrial invasion
MRI: Magnetic resonance imaging
NEEC: Non-endometrioid endometrial carcinoma
PACS: Picture Archiving and Communication System
PET/CT: Positron emission tomography computed tomography
SLN: Sentinel lymph node
SUV: Standardized uptake value

## Declarations

### Guarantor

The scientific guarantor of this publication is Ingfrid S. Haldorsen.

### Statistics and biometry

One of the authors has significant statistical expertise.

### Study subjects or cohort overlap

Some study subjects have been previously reported in:

1. Fasmer KE, Gulati A, Dybvik JA, et al (2020) Preoperative 18F-FDG PET/CT tumor markers outperform MRI-based markers for the prediction of lymph node metastases in primary endometrial cancer. Eur Radiol 30:2443–2453. 10.1007/s00330-019-06622-w

2. Ytre-Hauge S, Husby JA, Magnussen IJ, et al (2015) Preoperative tumor size at MRI predicts deep myometrial invasion, lymph node metastases, and patient outcome in endometrial carcinomas. Int J Gynecol Cancer 25:459–466. 10.1097/IGC.0000000000000367

3. Husby JA, Reitan BC, Biermann M, et al (2015) Metabolic Tumor Volume on 18F-FDG PET/CT Improves Preoperative Identification of High-Risk Endometrial Carcinoma Patients. J Nucl Med 56:1191–1198. 10.2967/jnumed.115.159913

### Methodology

• retrospective

• observational

• performed at one institution
