# Supplementary material for: Preoperative pelvic MRI and 2-[18F]FDG PET/CT for lymph node staging and prognostication in endometrial cancer—time to revisit current imaging guidelines?
Source: Eur Radiol. 2022 Jun 28;33(1):221–32. doi: 10.1007/s00330-022-08949-3 (PMC9755079; doi:10.1007/s00330-022-08949-3)
Supplement: Supplementary file 1 — (DOCX 442 kb) [file 330_2022_8949_MOESM1_ESM.docx]

Supplementary material

| **Supplemantary Table S1**  Clinical- and surgicopathological characteristics of patients included in the study cohort (n=361), compared with patients in the entire prospective cohort (n=625) diagnosed during October 2011 to July 2019. | | |
| --- | --- | --- |
|  | Study cohort (n=361) | Prospective cohort (n=625) |
| Age (median, range) | 68 (30, 90) | 68 (27, 91) |
| Preoperative histology^a^, n (%) |  |  |
| EEC G1–G2 (low-risk) | 240 (66) | 381 (62) |
| EEC G3+NEEC (high-risk) | 121 (34) | 232 (38) |
| MI^b^, n (%) |  |  |
| < 50% | 212 (60) | 346 (58) |
| ≥ 50% | 144 (40) | 251 (42) |
| CI^b^, n (%) |  |  |
| no | 308 (86) | 507 (84) |
| yes | 51 (14) | 95 (16) |
| Lymph node surgery, n (%) |  |  |
| no | 141 (39) | 268 (43) |
| yes | 220 (61) | 356 (57) |
| LNM^b^, n (%) |  |  |
| no | 193 (88) | 308 (85) |
| yes | 27 (12) | 53 (15) |
| Histologic subtype^b^, n (%) |  |  |
| EEC (G1–G3) | 285 (79) | 474 (76) |
| NEEC | 76 (21) | 151 (24) |
| Histologic grade (EEC only)^b^, n (%) |  |  |
| G1–G2 | 240 (85) | 390 (83) |
| G3 | 43 (15) | 78 (17) |
| FIGO stage^b^, n (%) |  |  |
| I | 283 (78) | 453 (73) |
| II | 33 (9) | 51 (8) |
| III | 34 (9) | 79 (13) |
| IV | 11 (3) | 41 (7) |
| Adjuvant therapy, n (%) |  |  |
| no | 230 (64) | 379 (61) |
| yes | 131 (36) | 245 (39) |
| CI, cervical stroma invasion; MI, myometrial invasion; EEC, endometrioid endometrial carcinoma, G, grade; NEEC, non-endometrioid endometrial carcinoma; FIGO, The International Federation of Gynecology and Obstetrics System; LNM, lymph node metastases.  ^a^Based on preoperative biopsy from curettage/pipelle.  ^b^Based on final histopathology after surgical staging.  Missing data (numbers): Preoperative histology from biopsy (12), MI (28), CI (23), LN surgery (1), Histologic grade (6), FIGO (1), Adjuvant therapy (1). | | |

| **Supplementary Table S2**  Clinical- and surgicopathological characteristics of 220 EC patient with both preoperative histology risk status, pelvic MRI, [^18^F]FDG PET/CT, and surgical LN staging | |
| --- | --- |
| Age (median, range) | 69 (40, 88) |
| Preoperative histology^a^, n (%) |  |
| EEC G1-G2 (low-risk) | 115 (52) |
| EEC G3+NEEC (high-risk) | 105 (48) |
| MI^b^, n (%) |  |
| <50% | 116 (53) |
| ≥50% | 104 (47) |
| CI^b^, n (%) |  |
| no | 178 (81) |
| yes | 42 (19) |
| LNM^b^, n (%) |  |
| no | 193 (88) |
| yes | 27 (12) |
| Histologic subtype^b^, n (%) |  |
| EEC (G1-G3) | 154 (70) |
| NEEC | 66 (30) |
| Histologic grade (EEC only)^b^, n (%) |  |
| G1-G2 | 117 (53) |
| G3 | 102 (47) |
| FIGO stage^b^, n (%) |  |
| I-II | 186 (85) |
| III-IV | 34 (15) |
| Adjuvant therapy, n (%) |  |
| no | 107 (49) |
| yes^c^ | 113 (51) |
| CI, cervical stroma invasion; MI, myometrial invasion; EEC, endometrioid endometrial carcinoma, G, grade; NEEC, non-endometrioid endometrial carcinoma; FIGO, the international federation of gynecology and obstetrics system; LNM, lymph node metastases.  ^a^Based on preoperative biopsy from curettage/pipelle.  ^b^Based on final histopathology after surgical staging.  ^c^Chemotherapy (n=110), external radiation therapy (n=2) and hormonal treatment (n=1).  Missing data (numbers): Histologic grade (1). | |

| **Supplementary Table S3**  Lymph node (LN) surgery and prevalence of lymph node metastases (LNM) in all patients in the cohort (n=361), patients with low-risk (endometrioid G1–G2) preoperative histology (n=240) and patients with high-risk (endometrioid G3 or non-endometrioid (G3+NEEC)) preoperative histology (n=121) | | | |
| --- | --- | --- | --- |
|  | All patients (n=361) | Low-risk^a^ (G1–G2) (n=240) | High-risk^a^ (G3+NEEC) (n=121) |
| LN surgery, n (%) |  |  |  |
| no | 141 (39) | 125 (52) | 16 (13) |
| yes | 220 (61) | 115 (48) | 105 (87) |
| pelvic LN sampling | 141 (39) | 107 (45) | 34 (28) |
| pelvic+paraaortic LN sampling | 79 (22) | 8 (3) | 71 (59) |
| LNM^b^, n (%) |  |  |  |
| no | 193 (88) | 105 (91) | 88 (84) |
| yes | 27 (12) | 10 (9) | 17 (16) |

G, grade; NEEC, non-endometrioid endometrial carcinoma

^a^Based on preoperative biopsy from curettage/pipelle

^b^Based on final histopathology after surgical staging

**
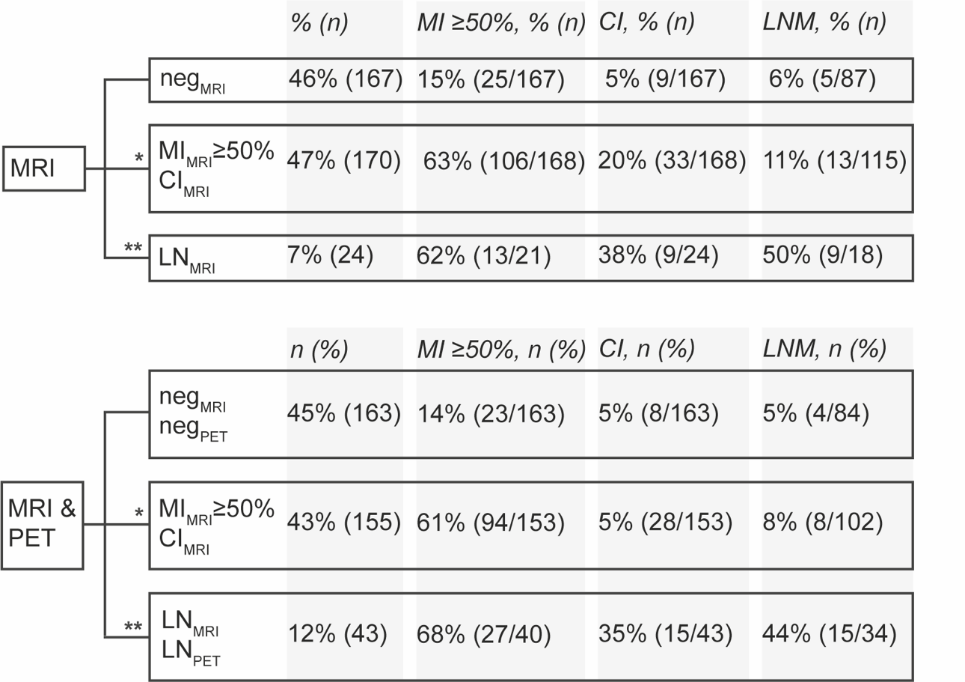
**

**Supplementary Figure S1**

Prevalence of histopathological deep myometrial invasion (MI ≥ 50%), cervical invasion (CI) and lymph node metastases (LNM) in 361 patients with endometrial cancer. Patients are divided into subgroups based on imaging findings from pelvic MRI alone (upper panels) and from combined MRI and [^18^F]FDG PET/CT (lower panels). Pelvic lymph node sampling was conducted in 220/361 patients.

MRI findings: MI_MRI_ ≥ 50%, deep myometrial invasion; CI_MRI_, cervical stroma invasion; LN_MRI_, enlarged (≥ 10mm) pelvic lymph node(s); neg_MRI_, MI_MRI_ < 50%, no CI_MRI_ and no LN_MRI_.

[^18^F]FDG PET/CT findings: LN_PET_, elevated [^18^F]FDG uptake in lymph node(s); neg_PET_, not elevated [^18^F]FDG uptake in lymph node(s)

Missing data (numbers): MI (7), CI (2)

*This subgroup includes patients with either MI_MRI_ ≥ 50% or CI_MRI_, but negative lymph node findings on MRI (upper panel) or on both MRI and [^18^F]FDG PET/CT (lower panel)

**This subgroup includes patients with positive lymph node findings on MRI (upper panel), or on both MRI and [^18^F]FDG PET/CT (lower panel)


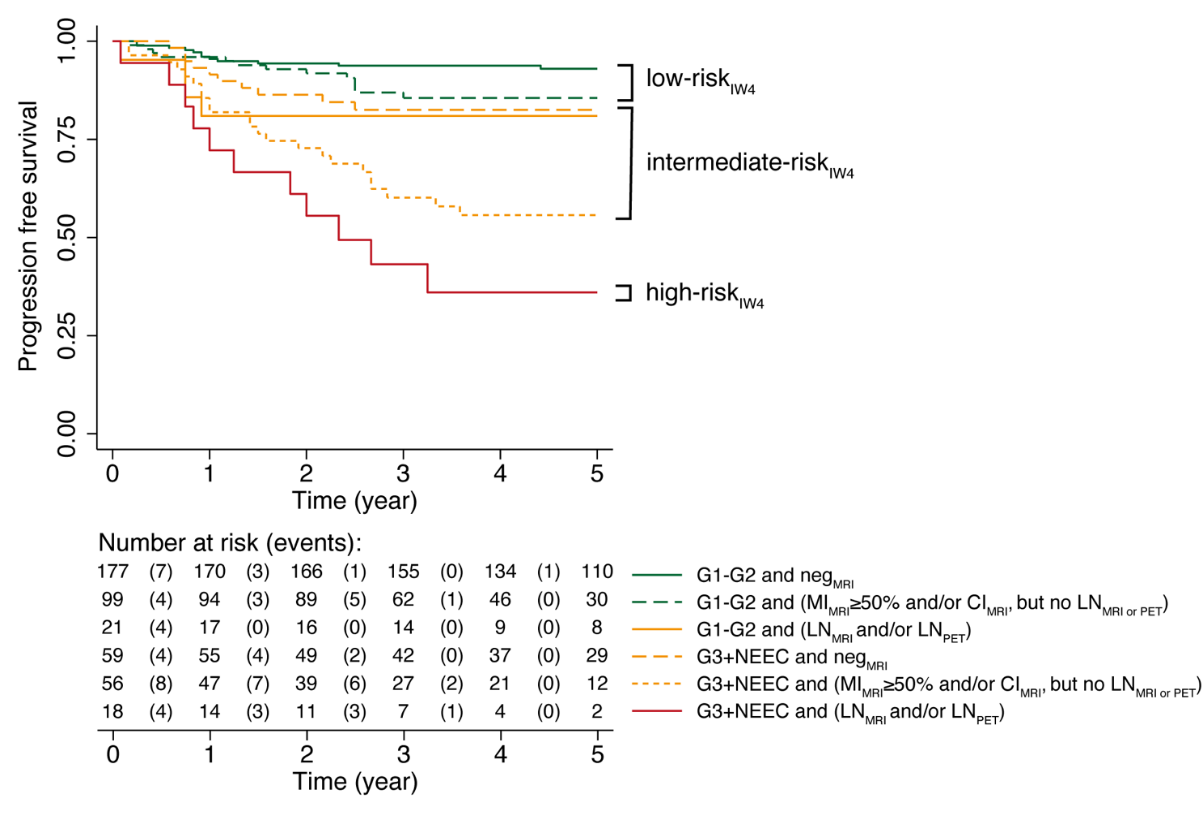


**Supplementary Figure S2**

Kaplan-Meier curves depicting progression-free survival for groups based on combined preoperative histological- and imaging assessments (MRI with selective [^18^F]FDG PET/CT if high-risk MRI findings) using imaging workup 4 (IW4).

Preoperative histology based on biopsy from curettage/pipelle : endometrioid grade 1–2 (G1–G2); endometrioid grade 3 or non-endometrioid (G3+NEEC).

MRI findings: MI_MRI_ ≥ 50%, deep myometrial invasion; CI_MRI_, cervical stroma invasion; LN_MRI_, enlarged (≥ 10mm) pelvic lymph node(s); neg_MRI_, MI_MRI_ < 50%, no CI_MRI_ and no LN_MRI_.

[^18^F]FDG PET/CT findings: LN_PET_, elevated tracer uptake in lymph node(s).
